# Mr. Bartley, on Quackery

**Published:** 1800-09

**Authors:** O. W. Bartley

**Affiliations:** Surgeon. Bradford


### *To the Editors of the Medical and Physical Journal.*

——— Exempta juvat spinis e pluribus una. HOR.

GENTLEMEN,

ON perusing the Observations of Mr. DAVID UWINS, in NO. XVI. of your truly valuable Miscellany, I was prompted to enlarge on the paper you honoured me by inserting in the preceding number; but my professional avocations prevented the execution of my intention during the course of the last two months. Leisure now permits me to make a few cursory remarks, which, should you indulge with a place in your Journal, will be thankfully esteemed by me, as an additional favour to those already conferred by your attention.

That gentleman observes, that " he suffered a degree of disappointment in finding no additional matter to what had been before advanced on the subject, excepting the publication of a few cases, which were by no means necessary to strengthen the evidence against all kinds of nostrums and specifics." I cannot, in the first instance, readily conceive the cause of that gentleman's disappointment. He acknowledges, that he observed in the table of contents, a paper " On the pernicious Effects of Quackery." I believe that paper, on perusal, he found correspond with its title. Had the table announced a communication *on the most effectual means of exterminating Quackery*, such an event would have been natural. He proceeds to assert, that those Observations were unnecessary. In this also I cannot acquiesce. Mr. U. previously observes, that "the subject had been for some time in a state of dormancy." It follows, therefore, that I was justifiable in the attempt at its resuscitation. It appears, its revival was necessary to call forth the latent opinions of this gentleman on the subject, (which were certainly ingenious and judicious). I am, however, inclined to prosecute further a matter of so much importance, and probably in a more explanatory manner. I do not, as insinuated, presume to assert, that a "neglect of legislative interference is the basis of the evil in question: By no means. When I hinted that such an interference was necessary, I did not conceive it probable that it could be exerted towards its total suppression; whilst the revenue is benefitted by the advantages accruing from the permission of patent medicines, such an event is scarcely to be hoped for. My plan, I flatter myself, is not so chimerical; its utility I submit to your superior judgment. For mine own part, I do not conceive a total subjugation of these vaunting empirics necessary. If I can contribute to the security of the welfare of my countrymen, my aim is accomplished. — I would have a law enacted, that if any person has a recipe for which he wishes to procure a patent, (whether his own invention or communicated to him by others) the nostrum should be first of all submitted to the inspection and analyzation of a committee of professional men, established for that purpose. If, after duly investigating its component parts, they should be of opinion that its administration would be serviceable in any particular complaint, to such disease alone should it be appropriated; and if, thus authorized, he obtain a patent, he should incur a heavy fine, should he at any time advertize the same nostrum as a remedy in any other disorder. By these means, although from political motives an evil is tolerated, it will be disarmed of its power to do mischief.

Mr. Custance observes, (in no. xvii.) that cases designed to evince the dangerous effects of Quackery to its deluded followers, should be communicated to the public through the same channel as the doctors' advertisements. He will permit me here to disagree with him. So marked an attention to the machinations of these worthless miscreants, would be too great a condescension on the part of the Faculty, and would ultimately conduce to their advantage; for these wily pests would find little difficulty in persuading their credulous victims, that the super-eminence of their characters, and the astonishing efficacy of their medicines, had attracted the envy of the profession, who were obliged to adopt that method to depreciate its value. These insinuations would be readily swallowed by such persons as are usually operated on by advertisements of that nature.

Thus,

Thus, in attempting to leſſen their credit, we ſhould, on the contrary, ſtrengthen their power, and furniſh them with materials to purſue their deſtructive career.

I ſhall not proſecute this ſubject further at preſent, but hope that thoſe gentlemen who have already given their opinions on the ſubject will with freedom reject, improve, or enlarge on the plan I have hinted thus lightly, or ſuggeſt other means more prudent or practicable.

I am induced from lately obſerving the frequent diſputes among ſeveral of your correſpondents, reſpecting the extraction of the placenta, (without hazarding an opinion myſelf) to ſtate two caſes which occurred to me not long ſince.

On Sunday, April 20, I was called to Elizabeth Baker, of Weſtwood, a ſmall village near this town, to attend in delivery. When I arrived, I found her ſtanding with her back reſting againſt the wall; and learned from the attendant women, on enquiry, that ſhe had been in this erect poſition for ſome hours. Not being able to ſit or even lie down, without the greateſt inconvenience, I concluded at the moment, from this information, that delivery was very near; I therefore placed her gently on the bed, and proceeded to examination. On attempting to introduce my fingers, I felt ſome reſiſtance; and examining further, I found that the uterus and its contents were ſo far preſſed into the vagina, that ſome part protruded a little through the os externum; a pain which immediately followed, would have forcibly expelled it, had not I made a conſiderable reſiſtance with my hand. The os tincæ not being dilated to the compaſs of a ſhilling, I was convinced that delivery was for the preſent impracticable; I therefore attempted to return the uterus, and happily ſucceeded; and a copious evacuation of urine ſhortly ſupervened, which diſcharge had been ſuppreſſed nearly two days by the preſſure on the meatus urinarius. I directed that ſhe might be conſtantly kept in a horizontal poſition, and the bowels moderately open by occaſional emollient clyſters, an anodyne being now and then introduced. She thus continued a week without a recurrence of pain, prolapſe of the uterus, or impediment in the alvine or urinary evacuations.

On Monday the 28th, I was again called, about one o'clock in the morning. On arriving, I found the child had been delivered without aſſiſtance, about three-quarters of an hour; that conſiderable hæmorrhage had enſued, but had then ceaſed; and the placenta was not yet extracted. The woman being extremely weak, and conſiderably exhauſted by the effuſion of blood, I thought proper, as the hæmorrhage had ſubſided, to wait wait fome time before I attempted its extraction: But fome flight pains occurring, (which I hoped would effect its expulfion without difficulty) I made fome efforts to bring it away, by gently drawing the funis; but in vain. On introducing my hand, I found a morbid adhefion had taken place near the fundus uteri. As the uterus had not much contracted, I defifted from further endeavours, confidering the weaknefs of the patient, whofe fituation was rather perilous from recent exhauftion; her lips livid, pulfe fmall, and her whole countenance pale and ghaftly. I determined to leave its expulfion to the efforts of Nature, and waited with patience about four hours, contenting myfelf with gently ftraining the funis at the recurrence of every pain. By this time the patient became very importunate in her requeft to have it taken away at all events, being extremely anxious to be put into bed; I therefore, in compliance, again introduced my hand, and removed feveral large clots of blood which obftructed its paffage; then cautioufly introducing my fingers round the whole fubftance of the placenta, between it and the uterus, I gradually detached and brought it away entire. Slight hæmorrhage fucceeded, and repeated faintings, which were fo alarming that I began to wifh I had perfifted in my original refolution of trufting the operation to the efforts of Nature, which I doubt not would have been fufficient, had I not acceded to her impatience. By adminiftering cordial, tonic, and volatile draughts frequently, fhe foon recovered, and was, in the courfe of a fortnight, able to leave her room without material injury or inconvenience.

The fecond cafe is of Elizabeth Munday, refiding at Avonclift, near this town. I was called on to attend her in labour, May 16. The prefentation of the child was natural, and nothing material occurred prior to delivery, which happened foon after my arrival. I did not intend to proceed to the extraction of the placenta until Nature evinced by her efforts for its expulfion, that my affiftance was neceffary. But about ten minutes after the birth of the child, a hæmorrhage enfued, which in a fhort time became fo profufe as to greatly endanger the life of my patient. I then judged it expedient to attempt its delivery without further delay. I took hold of the funis, which was of a texture uncommonly flight, and drew it in the genteleft manner; but unfortunately, notwithftanding I proceeded with the utmoft caution, it feparated at the placenta. Although fuch an accident was new to me, I did not fuffer alarm to conquer my circumfpection. I cautioufly introduced my hand into the uterus; and, as in the preceding cafe, gradually detached the placenta, which I brought away entire. Hæmorrhage ftill continued, and I became extremely apprehenfive for my patient's tient's ſafety; but happily, by the application of wet cloths, &c. and ſupporting her occaſionally with cordials, I ſucceeded in removing every alarming ſymptom, and ſhe is now tolerably ſtrong and healthy.

The former of theſe caſes may tend to exemplify, that the placenta may be retained with ſafety a much longer time than is generally admitted; and the latter, that although a forcible extraction is ſometimes neceſſary from accident, it may be eaſily effected without the neceſſity of thruſting the fingers into the ſubſtance of the placenta.

Before I conclude, I ſhall further intrude on your patience by calling your attention to another ſubject. In the firſt Vol. of your Miſcellany, p. 181, Profeſſor Hufeland, of Jena, recommends the uſe of artificial muſk in the hooping cough. I have made ſeveral eſſays of the virtues of that compoſition, and find it efficacious not only in that complaint but in ſeveral others, a few of which I ſhall briefly ſtate to you. I generally adminiſter it in the form of a tincture, thus prepared: Two drachms of the reſinous extract, obtained by the proceſs there recommended, I diſſolved in eight ounces of alcohol, which formed a tincture of a paliſh yellow colour, and afforded a very grateful ſmell. Two ſons of a gentleman at Briſtol, the one about nine years of age, and the other ſix, were afflicted with the hooping cough. In the eldeſt, an epiſtaxis conſtantly attended the paroxyſms of coughing, accompanied with ſevere pains in the region of the thorax. The diſorder in both ſeemed to reſiſt the power of the remedies uſed. Emetics, &c. were adminiſtered by a medical gentleman there reſident, but without any viſible good effect. Having buſineſs that called me to that city, I accidentally ſaw the children, and adviſed their friends to make trial of this ſpecific, which they agreed to. I ſent a phial, containing about ℥jſs, directing the eldeſt to take twelve and the youngeſt ten drops of the tincture three times a day, in a tea-cup full of barley-water. I have, a few days ſince, received an account of its good effects, in a letter from my friend, a ſhort extract from which I ſhall here tranſcribe. "The bottle of drops you ſent me for John, has perfectly cured him of the hooping cough; on taking it, he immediately found relief, as it removed the pains he complained of about his cheſt, and ſtopped the bleeding of his noſe. Theſe drops, without the aid of any other medicine, have perfectly reſtored him. The youngeſt is alſo nearly recovered."——In two or three other inſtances I have alſo witneſſed its good effects; and am of opinion, that in this complaint its efficacy ſtands unrivalled.

In

In a cafe of Diabetes Mellitus, its adminiftration was attended with fingular advantage. It occurred in an elderly man, who refides at Broughton, near Melkfham, in this county.—The concomitant fymptoms fufficiently characterifed the difeafe: Great emaciation, lofs of ftrength and appetite, with a flight degree of hectic. The difcharge of urine was very confiderable, and varying in its appearance, fometimes affuming that of whey, at other times (except when held in a glafs to the light) perfectly limpid; the tafte and fmell were fuch as are commonly diftinguifhable in that diforder. I gave him a confiderable quantity of hepatifed ammonia, recommended alkaline drinks, and a total abftinence from vegetable food and fermented liquors. The difeafe did not, however, feem difpofed to yield to this mode of treatment; on the contrary, the fymptoms became daily more formidable. Confidering his cafe hopelefs, and, I confefs, with no very fanguine expectations of fuccefs, I determined on a trial of the tincture before defcribed. I directed him to take 25 drops bis terve die, perfifting in the fame regimen I before advifed, and omitting the hepatifed ammonia. I was agreeably furprifed by his fpeedy amendment, and at length his complete recovery. From the time he commenced taking the drops, the alarming fymptoms gradually fubfided, his appetite returned, his ftrength became by degrees repaired, the urinary evacuations became lefs frequent, never involuntary, divefted of its faccharine tafte and peculiar fmell, and its appearance healthy; in fine, he foon found himfelf in a ftate of complete convalefcence. I have found it a powerful tonic and corroborant in nervous diforders, and cafes of extreme debility; feveral of which, would time permit, I could now lay before you. This, however, I muft defer till a future opportunity; I fear I have already trefpaffed too long on your patience, for which I folicit your excufe. Accept my warmeft wifhes for the profperity of your excellent and laudable undertaking; and believe me to be, with refpect,

Gentlemen,

Your moft obedient fervant,

*Bradford, Aug.* 9, 1800. O. W. BARTLEY, Surgeon.

